# Systemic Metabolism, Its Regulators, and Cancer: Past Mistakes and Future Potential

**DOI:** 10.3389/fendo.2019.00065

**Published:** 2019-02-12

**Authors:** Jeff M. P. Holly, Kalina Biernacka, Claire M. Perks

**Affiliations:** Faculty of Medicine, School of Translational Health Science, University of Bristol, Southmead Hospital, Bristol, United Kingdom

**Keywords:** cancer, metabolism, diabetes, obesity, lifestyle, prevention, post-genomic

## Abstract

There has been a resurgence of interest in cancer metabolism; primarily in the resetting of metabolism within malignant cells. Metabolism within cells has always been a tightly regulated process; initially in protozoans due to metabolic enzymes, and the intracellular signaling pathways that regulate these, being directly sensitive to the availability of nutrients. With the evolution of metazoans many of these controls had been overlaid by extra-cellular regulators that ensured coordinated regulation of metabolism within the community of cells that comprised the organism. Central to these systemic regulators is the insulin/insulin-like growth factor (IGF) system that throughout evolution has integrated the control of tissue growth with metabolic status. Oncological interest in the main systemic metabolic regulators greatly subsided when pharmaceutical strategies designed to treat cancers failed in the clinic. During the same period, however the explosion of new information from genetics has revealed the complexity and heterogeneity of advanced cancers and helped explain the problems of managing cancer when it reaches such a stage. Evidence has also accumulated implying that the setting of the internal environment determines whether cancers progress to advanced disease and metabolic status is clearly an important component of this local ecology. We are in the midst of an epidemic of metabolic disorders and there is considerable research into strategies for controlling metabolism. Integrating these new streams of information suggests new possibilities for cancer prevention; both primary and secondary.

## Introduction

The revolution in genetics has led to considerable advances in our understanding of the complexity and plasticity of clinical cancers: providing new insight into the problems of treating advanced cancers (1). A realization of the major challenges facing current treatment strategies for the medical management of advanced disease has reinvigorated attention on prevention. With the gradual decline in tobacco usage across North America and Western Europe surveys of public health trends predict that smoking as the leading modifiable cause of cancer will soon be overtaken by metabolic-imbalance, caused by overconsumption of calories and lack of physical activity and with the obvious clinical sequelae of obesity and type 2 diabetes (2). This has accelerated interest in the role that key metabolic regulators play in cancer. Considerable experimental work in cell and animal models indicated that the insulin/IGF system has important effects on cancer; this converged with evidence from population epidemiology such that by the turn of the century virtually the whole pharmaceutical industry was developing anti-cancer drugs targeting this system (3). Over the subsequent years this excitement has largely waned as each company introduced drugs into clinical trials with disappointing results and subsequent withdrawal. However, over the same period the explosion of information from cancer genetics has changed our understanding of cancer etiology and this review aims to revisit the potential role of metabolic regulation and insulin/IGFs in the common epithelial cancers in the light of this new understanding. Firstly, we will describe the insulin/IGF system and how it has evolved in a pivotal role for the integration of tissue growth with metabolic status. Their well-established role in synchronizing metabolic regulation according to nutritional status will be briefly discussed in relation to its relevance to cancers. The evidence linking insulin/IGFs with cancer has been extensively reviewed elsewhere in recent years (4–6) and therefore we will limit the discussion here to a few comments. We will then outline how the flaws in the prevailing genetic paradigm for cancer etiology have been exposed by recent findings and how this should alter our perceptions of the actual events that determine the course of these cancers. This will then set the context of how, and at what stage, metabolic status and its regulators, including insulin/IGFs, may be affecting the course of carcinogenesis.

The evidence linking metabolic disturbance (and insulin/IGFs) with cancer added to the shift toward considering prevention as a viable option. At the same time evidence was emerging indicating that by the time cancers were clinically evident the heterogeneity and plasticity within the tumors was so complex that it presented considerable challenges to treatment. This stirred many leading cancer researchers to consider that it was timely for a redirection of efforts away from cures and more toward prevention (1). It now seems a little incongruous that so much effort was then directed toward developing treatments targeting the IGF-system that were then tested in clinical trials in patients with advanced cancer. At the time however it was a popular strategy within the pharmaceutical industry and it built on some early successes in targeting other growth factors. It should however be noted that no other targeted therapy has ever worked for any cancer when applied to all patients, without some form of patient selection, usually based on biomarkers that indicated a responsive subpopulation. In the absence of proven markers of therapeutic response, agents targeting the IGF-system have thus far only been tested in unselected patients (5, 7). With hindsight this was a mistake and the poor results could maybe have been predicted. We will then end by discussing how we should move beyond these disappointments with some thoughts on the potential of where research into the insulin/IGF-system may impact on future strategies to combat cancer.

## The Insulin/IGF System

The insulin/IGFs form part of a fundamental regulatory system that comprises four secreted peptides; two with very restricted expression that are just secreted from specialist endocrine cells in the pancreas, proinsulin and insulin, and two more general signals secreted from most cells throughout the body, IGF-I, and IGF-II. These peptides are very homologous and originated by gene duplications and indeed the IGF-II gene is adjacent to the insulin gene. The peptides also share considerable functional homology. It is often forgotten that in addition to being discovered as growth factors that mediate the effects of growth hormone (GH) (8) they were also independently discovered as non-suppressible insulin-like activity (NSILA), distinguishable from actual insulin by specific antibodies; indeed in some bioassays insulin itself accounted for < 10% of the metabolic insulin-activity present in normal human serum (9). Following the structural characterization of these peptides it was realized that IGF-II was responsible for the majority of this metabolic “insulin-like” activity present in serum. In light of the similarity between these peptides, both structurally and functionally, their physiology has to be interpreted with an understanding of their relative abundance throughout the body: with IGF-II levels normally being 3- to 4-fold higher than that of IGF-I and around 1,000-fold higher than that of insulin (10). When considering the role of IGFs in cancer, the main focus is generally on their activity in promoting cell growth and survival and their major contribution to metabolic regulation is generally overlooked.

**Figure 1:**
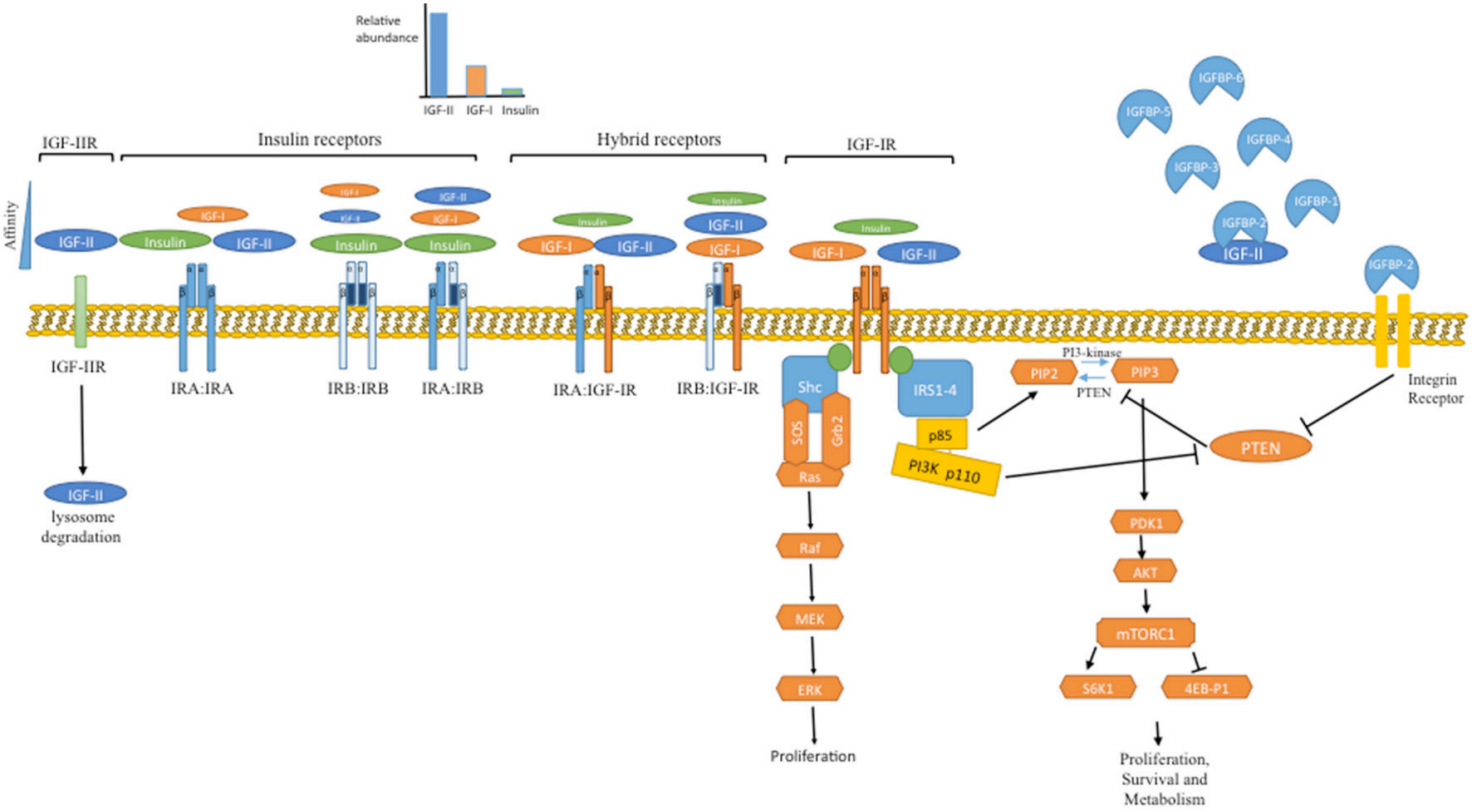
Overview of the IGF system. In the body the IGFs are constantly present at very high levels due to their binding to the IGFBPs, with IGF-II levels 3 to 4-fold higher than IGF-I. Following meals insulin is secreted from the pancreas but is the rapidly cleared, resulting in episodic transient exposure to the tissues. At the cellular level these ligands interact with a family of signaling tyrosine kinase receptors: the IGF-IR and the insulin receptor, which exists in two alternatively spliced isoforms. The IRB predominantly only binds insulin whereas the IRA also binds IGF-II, which due to its abundance is the most likely ligand. These receptors initiate intracellular signaling, principally the PI3K/Akt and Ras/Raf/MAP kinase pathways, that set in train processes that culminate in increased cell proliferation, survival and metabolism. In addition to maintaining levels of IGFs and restricting their interaction with cell surface receptors; the IGFBPs also have independent actions. Via interaction with integrin receptors IGFBP-2 suppresses PTEN, the phosphatase that counteracts the activity of PI3K and therefore IGFBP-2 can release the brake on IGF signaling. In addition to IGFBPs controlling the availability of IGFs there is also a cell surface IGF-IIR that limits the availability of IGF-II by targeting it for lysosomal degradation.

These ligands regulate cell functions via activation of classical tyrosine kinase cell surface receptors that, as with the ligands, share similar homology both structurally and functionally (11) (Figure 1). The IGF-I and insulin receptors are all heterotetramers: both genes are translated and the mature protein is cleaved to yield an extracellular α-subunit and a disulphide-linked transmembrane β-subunit and these dimerise to form the tetramer. The IGF-IR binds IGF-I and IGF-II with high affinity and has little affinity for insulin. The insulin receptor exists as two isoforms, due to alternative mRNA splicing with the IR-A isoform containing 12 fewer amino acids in the extracellular C-terminal domain of the α-subunit due to splicing out of exon 11 whereas the IR-B isoform has these additional amino acids due to exon 11 being spliced in. These extracellular α-subunits form the ligand binding domain. While the IR-B predominantly binds insulin and has a much lower affinity for IGF-I/-II and hence is the classic insulin receptor; the loss of 12 amino from IR-A diminishes this specificity and results in a relative increase in affinity for IGF-II (12). As a consequence IGF-II binds IR-A with an affinity approaching that of insulin, and IR-A also binds proinsulin with high affinity, in contrast to the very low affinity for these ligands with IR-B (12). Although IR-A binds insulin and IGF-II with similar affinities there is evidence that these ligands induce differential signaling and effects (13, 14). This has important pathophysiological implications, especially in view of the marked differences in abundance of these ligands. The α-/β- dimers of the insulin and IGF-IR are so similar that they hetero-dimerise to form hybrid receptors, both IR-A/IGF-IR hybrids and IR-B/IGF-IR hybrids, depending on the relative expression of each receptor in a particular cell. These hybrid receptors appear to predominantly act as IGF-I receptors (15, 16), but their pathophysiology is still poorly understood.

There is also an IGF-II receptor (IGF-IIR) that is completely unrelated and is structurally and functionally different from the other IGF receptors and is a single large transmembrane protein (17, 18) The IGF-II receptor binds IGF-II with very high affinity and has very little affinity for IGF-I or insulin but is generally considered to not act as a traditional signaling receptor in response to IGF-II binding. It is thought to act as a clearance receptor for IGF-II controlling cell exposure to IGF-II, internalizing and directing IGF-II to lysosomes for degradation; since disruption of gene expression in mice resulted in elevated IGF-II levels and overgrowth (19). The cellular location of the IGF-IIR is dynamically regulated by insulin in the same way as the glucose transporter, Glut 4 (20–22); these receptors are mainly intracellular but are rapidly translocated to the cell surface in response to insulin. This dynamic translocation of these receptors has been known for over 30 years and although still poorly understood it could, like the dynamic translocation of Glut4, potentially represent a novel means for metabolic control, although direct evidence for this has yet to be demonstrated. When nutrient abundance triggers pancreatic insulin release, the insulin could result in internalization of the IGF-IIR that could then lead to more IGF-II interacting with the IGF-IR and the IR-A complimenting the direct actions of insulin itself; however this is yet to be proven experimentally. These IGF-II receptors are in addition, clearly multifunctional: their most well characterized role is as mannose 6-phosphate receptors involved in the targeting of lysosomal enzymes to the lysosomes within the cell. These receptors however, also bind latent transforming growth factor-β (TGF-β) and enable its activation on the cell surface. They also bind to retinoids, urokinase-receptors, and many other proteins. As TGF-β has many important effects on cancers, particularly in relation to differentiation status and stimulating epithelial to mesenchymal transition (EMT) this interaction may have potential implications for interactions between the insulin/IGF-system and these other regulatory pathways. The functional consequences of interactions between IGF-II and the other possible ligands of the IGF-II receptor are far from clear (17, 18, 23).

## Insulin and IGF: Similar but Very Different Regulatory Systems

Insulin, the IGFs and their receptors clearly evolved from early gene-duplication events; yet despite their structural similarities and the similarities of their cellular activity, they have evolved to operate as very different regulatory systems. Insulin is a classical endocrine regulator; its expression is very restricted, principally just to the beta-cells of the pancreatic islets where there are intracellular stores in secretory granules and it is secreted via the regulated secretory pathway in response to stimuli, principally glucose fluctuations that enables an acute regulation of metabolic activity. Insulin is secreted in an episodic manner from the pancreas into the circulation; blood levels therefore fluctuate widely, and it is transported in the blood to target tissues where it activates cell receptors. In contrast the IGFs are expressed in most cell types in tissues throughout the body and they are secreted via the constitutive secretory pathway: they are not stored in intracellular secretory granules, but they are secreted immediately, as soon as they are produced (10). Although there are no intracellular stores of IGFs a sophisticated system has evolved for maintaining extracellular reservoirs of IGFs formed due to their association with very specific high affinity binding proteins.

In humans there are 6 binding proteins (IGFBP-1 to −6) that bind IGFs with high affinity but do not bind insulin; these are unrelated to the cell-surface receptors but are structurally very closely related to each other (24). The six IGFBPs all have very distinct functional properties and they are produced in different quantities and combinations in different tissues (24). The IGFBPs sequester the IGFs as they are secreted and considerably slow their clearance; enabling very high concentrations of IGFs to build-up. In the circulation two of the IGFBPs, IGFBP-3, and IGFBP-5 are bound to a further large glycoprotein, the acid labile subunit (ALS) that is present in excess. This ternary complex is too large to cross capillaries and hence is retained in the circulation and further slows clearance such that in adult humans the total IGF-I and IGF-II concentration in the circulation is around 100 nanomolar. This is around 1,000 times higher concentration than that of insulin and while insulin levels fluctuate acutely in response to metabolic requirements the circulating levels of IGFs are very stable due to the very long half-life of these complexes (10).

Although expressed in most tissues, the majority of the IGFs present in the circulation originate from the liver where the production of IGF-I and IGFBP-3 are regulated by GH but are also very dependent on nutritional intake (25). The high, stable levels of circulating IGFs therefore provide a large pool of metabolic regulators that reflect chronic nutritional status. At the cellular level, optimal activation of the insulin and IGF receptors are both achieved with just 1–2 nanomolar concentrations, indicating that there is a vast excess of IGFs in the circulation. Hence while the activity of insulin throughout the body is largely determined by the rate of secretion from the pancreas, in contrast the constitutive secretion of the IGFs within any tissue is just one of the determinants of the total amount of IGF that the cells are exposed to and the control of activity is much more complex (10, 24). The IGFs bind the IGFBPs with affinities higher than that of the IGF-I receptor, so most of the IGF in the body has restricted availability for receptor activation. Activity in a tissue is therefore not necessarily determined by secretion rate of IGFs and not necessarily determined by total IGF concentration (10).

The IGFs are made available from the large soluble stores, maintained due to their association with the high affinity IGFBPs, in a controlled manner by different mechanisms that lower the affinity of these interactions: shifting the equilibrium in favor of IGFs binding to cellular receptors. In the circulation the majority of IGF is associated with IGFBP-3 and the ALS. The ALS binds to a C-terminal region of IGFBP-3 that also binds to proteoglycans present on cell surfaces and in the extracellular matrix (ECM). It is therefore possible that such proteoglycans on the surface of endothelial cells compete for binding to IGFBP-3 and displace the ALS generating a binary complex that can then perfuse across the capillaries into the tissues (26). There is however an additional more specific mechanism for controlling delivery of IGFs from the IGFBP-maintained reservoirs. Each of the IGFBPs appears to be subject to specific limited proteolysis (27). The proteolysis of IGFBPs lowers the affinity with which they bind IGFs, not always complete loss of binding, but even a small decrease in affinity could result in a shift in the complex equilibrium that must exist *in vivo* presumably making the IGFs more available for cell receptors. This physiology has important implications for cancers; tumors become life threatening when they invade and spread around the body, processes that depend on proteolytic degradation of the ECM. This can mobilize latent IGFs held with soluble IGFBPs or IGFBPs that are sequestered onto the ECM due to binding to proteoglycans. In addition the same ECM proteases can also act on the IGFBPs increasing the bio-availability of the large latent reservoir of IGFs that they hold (24).

## Intracellular Signaling and Metabolism

The IR and the IGF-IR are members of the tyrosine kinase family of cell surface receptors; several homologous members of which are recognized to be oncogenes. Upon binding of insulin/IGFs to the α-subunit there is a conformational change resulting in activation of the tyrosine kinase activity within the intracellular β-subunit that results in autophosphorylation of several intracellular sites that then provide docking sites for the recruitment of a variety of adaptor proteins including the insulin receptor substrates (IRS-1 to−4), Shc and receptor for activated C kinase 1 (RACK1). This then enables the assembly of signaling complexes that activate networks of signaling pathways. The two best characterized of these are the PI3K/Akt/mTOR/S6K and Grb2/SOS/Ras/Raf/MAP kinase pathways (28, 29) (Figure 1). Of particular interest in relation to cancer metabolism is the PI3K pathway. In single cell organisms, such as yeast, PI3K acts as a nutrient sensor and is directly activated by the availability of amino acids with consequent activation of mTOR/S6K (30). In higher multicellular animals PI3K is not directly activated by nutrients but has evolved into heterodimers comprising a p110 catalytic subunit and a p85 regulatory subunit. In quiescent cells these dimers are cytoplasmic and the p85 subunit represses the catalytic activity of p110. Following activation of the IR or the IGF-IR these dimers are recruited to the cell membrane by IRS-1 and IRS-2 via SH2 domains in p85; the binding of which relieves the repression of p110 activity (31). This evolved system enables regulation according to nutrient availability to be integrated by communal signals across communities of cells rather than being controlled directly by nutrients at the individual cellular level, thus ensuring that growth and metabolism are synchronized within metazoans. The lipid kinase activity of PI3K, that recruits and activates Akt, is opposed by the lipid phosphatase PTEN (phosphatase and tensin homolog), a tumor suppressor gene, expression of which is commonly lost in many cancers (31). Interestingly the p85 regulatory subunit of PI3K also binds to PTEN resulting in enhanced phosphatase activity, in contrast to its repression of PI3K activity (32). Thus, p85 can negatively regulate the PI3K/Akt pathway by repressing p110 PI3K and enhancing PTEN. An additional layer of metabolic control also operates via IGFBP-2, itself metabolically regulated, which when free from IGFs can interact with cell surface integrin receptors and suppress PTEN activity (33). This appears to provide a synchronized communal control of the PI3K/Akt pathway with IGFs acting to apply the “accelerator” and IGFBP-2 also removing the “brake.”

## Insulin/IGFs, Nutrition, Metabolism, and Growth

Early human development is tightly regulated by the insulin/IGF axis (34), ensuring that growth and development only proceed when the cells receive the appropriate signal indicating that sufficient nutrients are available. *In utero* nutrients are supplied constantly via the placenta and not via the gut. Pancreatic insulin secretion is less dependent on acute variations in nutritional intake and by far the most abundant ligand for insulin/IGF receptors is IGF-II which appears to play an important role in placental function and in the control of nutrient partitioning (35). Both IGF-II and the IGF-II receptor are imprinted genes and it has been proposed that this has evolved to balance the genetic conflict between parents. The paternal imprinted genes enhance nutrient extraction from the mother and maximize the growth of the fetus to ensure the survival and development of the father's offspring; this is ensured by the imprinting of the IGF-II gene, with expression of just the paternal allele. In contrast it is in the interest of the mother to constrain fetal development and balance nutrient extraction to ensure her own survival and reproductive competence for future potential offspring, with potentially different fathers; this is effected by imprinted expression of the maternal IGF-IIR allele in the mouse, although this imprinting has not been conserved in humans (36). After birth the pituitary develops and takes control of the endocrine system and pituitary GH drives systemic IGF-I production, which then plays a more dominant role in growth and development. In rodents there is a clear switch at weaning when the expression of IGF-II is virtually ceased and there is an obvious end to the major systemic role that IGF-II plays. In higher mammals this switch does not occur and in humans IGF-II is the most prevalent IGF throughout life. In adult humans there are around 3- to 4-fold higher levels of IGF-II in the circulation, compared to IGF-I, and there is no abrupt end to the role of IGF-II as clearly happens in rodents. In rodent models there are therefore two fundamental differences in the IGF-II system with differences in the imprinting of the IGF-IIR and a virtual absence of IGF-II in the circulation of adult rodents but IGF-II remaining the most abundant of these peptides in adult humans. These experimental models therefore have limited value in investigating the role that IGF-II may play in adult cancers in humans. This major species difference has largely been overlooked in the use of these experimental models for examining interventions targeting the IGF-system for treating cancers; many agents worked well in the rodent models where IGF-I is predominantly the only ligand but when these were extended into clinical trials in humans the results have been disappointing (5, 7).

Ever since cancers were likened to embryonic rests or remnants (37), it has been commented that cancer cells appear to reactivate a program analogous to that active in early development. Increased cell proliferation, increased energy metabolism, evasion of growth suppressors, evasion of contact-inhibition, evasion of cell death, invasion, metastasis, and induction of angiogenesis are the hallmarks of cancer, but all of these processes are also normal and essential requirements for the development and morphogenesis that occur in early fetal life when IGF-II is the predominant metabolic regulator. These properties do not therefore have to be acquired individually by cancers but if the normal developmental program is reactivated then they are naturally integrated together. Part of this program, essential during early development (34), is that these processes are tightly coordinated according to nutrient availability. That the organism is in the fed state is signaled to the tissues via insulin and/or IGFs activating the PI3K pathway, ensuring that growth and development only proceeds when adequate fuels are available. As cancer cells reactivate a program analogous to that active in early development this fundamental control will also be engaged. In addition, these processes are maintained in some normal tissues throughout the lifespan: for example, the constant renewal of epithelial surfaces and the ability of epithelial surfaces to repair when wounded. Wound healing requires cell proliferation, migration, inflammation, angiogenesis: indeed it has been commented that tumors are analogous to wounds that do not heal (38) or even wounds that over-heal (39).

Nutrition fuels childhood growth but this is context dependent; if too much energy is expended by physical activity, maintaining temperature control or by stress, then growth can be completely arrested. In childhood such contextual changes permit nutrients/energy to be redirected to muscle for physical activity or shivering to keep warm or redirected from bone growth to essential functions such as brain activity and immune responses etc. Tumors reactivate the same pathways that are dependent on cues conveying nutrient/energy status, but these may also be context dependent. Other systemic disturbances such as insulin-resistance, inflammatory conditions, stress etc. can result in repartitioning of nutrients/energy that can potentially benefit the tumor.

During early embryonic development the IGFs, predominantly IGF-II, not only ensure that growth is co-ordinated with the availability nutrients and energy, but also promotes cell migration and invasion. Tissue morphogenesis requires pluripotent stem cells to differentiate into a variety of cell types to form a mature tissue and IGF-II maintains cell survival during these transitions of differentiation; as has been demonstrated for a number of cell lineages including the differentiation of myoblasts in skeletal muscle (40). The IGFs promote and help maintain the rapidly increasing cellular heterogeneity during tissue morphogenesis. It is likely that similar roles are reactivated in tumors. Indeed, this has been proposed, and some experimental evidence was generated to support the model, that IGF-II production by some clones within tumors may help maintain tumor heterogeneity (41). As extensive tumor heterogeneity has consistently been observed and is increasingly recognized as contributing to both cancer progression and failure of treatments, the support of such heterogeneity may be an important role for IGFs. With multiple clones it is increasingly likely that one will acquire the properties required for spread and growth in other tissues and also likely that some clones will survive therapeutic interventions.

## Acquisition of a Malignant Phenotype

In order to best assess how to reduce the burden of cancer we need to appreciate the factors contributing to this burden and how such considerations should be reassessed in light of the explosion of new findings. Cancer is indeed initiated by genetic aberrations that result in altered cell behavior leading to a breakdown in the homeostasis that is normally maintained by the social network of cells within tissues. This culminates in antisocial growth and the spread of colonies of cells that can threaten the viability of the organism. Advances in techniques for characterizing the genome have revealed the extent of the considerable genetic damage that accumulates within tumors. It is also however now evident that considerable genetic alteration also accumulates in normal tissues where constant cell renewal occurs and that this is a natural consequence of normal physiological aging (42–44). Every time that a cell divides the DNA is not copied with 100% accuracy and with cells that regularly divide considerable genetic damage accumulates normally over the years. This has raised the possibility that many, or even most, of the common age-related epithelial cancers could occur through this normal physiological process. The increasing prevalence with age of indolent cancers that can be detected at autopsy or by screening healthy populations is also consistent with the primary neoplastic initiation events being frequent natural occurrences and a consequence of normal aging.

Although cancer cells are considered to behave in a very abnormal manner: growing, invading and spreading; it has long been recognized that these are the very same processes that occur normally during embryonic development and throughout life in processes such as inflammation and wound healing. The concept that cancer cells may be embryonic rests was proposed in the nineteenth century (37). Such concepts have evolved into the realization that there are patterns of cell behavior that are programmed within all cells, but which are normally only expressed during embryogenesis or wound healing. These same processes can however be inappropriately reactivated in neoplastic cells, probably either in response to some form of stress or as the cell slips back down a gradient of differentiation status (45–47). Embryonic and early life development have been programmed throughout evolution such that they are tightly synchronized and tuned to environmental exposures, especially metabolic conditions. This developmental programming ensures that the organism learns to adapt to the nutritional/metabolic environment. This adaptation is conveyed to the large populations of cells within the organism by hormones, cytokines, and growth factors: the social signals that ensure that all cells within tissues act in a unified synchronized manner. There has been considerable interest that this early life adaptation has long-term consequences with many lines of research implicating early-life programming affecting the development of chronic diseases later in life as originally proposed by David Barker (48). Cancer cells, as they de-differentiate, reactivate many of the early life programs and it is increasingly evident that with this they also acquire similar cell plasticity and use the same coordinating cues from these fundamental cell regulators.

## Cancer Mutations and Heterogeneity: a Paradigm Shift

Although cancers arise from genetic damage they clearly do not arise from a mutation in a single gene or even in a small number of genes. Uncontrolled cell growth has always been a threat to complex organisms and, as mutations are an inevitable natural consequence of cell division, evolution has crafted many mechanisms to prevent such gene mutations from directly leading to lethal cancers (49).

Mutations contributing to cancer pathophysiology are termed driver mutations whereas those not currently known to contribute are termed passenger mutations. It was originally thought that for most cancers the initiation and progression was primarily due to a few common driver mutations but there is increasing awareness that there are also many additional plausible driver mutations that occur at low frequency or are idiosyncratic to an individual clone of cells. The malignant phenotype is then probably a result of the collaborative effect of these many mutations that could number many thousands for any particular tumor.

Gene mutations can occur from exposure to genotoxic agents, but they also occur naturally due to errors during DNA replication. Indeed errors in DNA replication occur naturally at a rate of around one in every 10^9^–10^10^ nucleotides per cell per division (50). Throughout life the cells forming the epithelial surfaces are continuously dividing in order to fulfill their normal function to replenish the epithelial barriers and this explains why around 90% of human cancers are carcinomas within epithelial tissues. In the human colon there are around 1–1.5 × 10^7^ crypts, each maintained by a small population of stem cells and it is estimated that these stem cells divide every 1–4 days. The rate at which mutations accumulate over the life-course is then a relatively simple calculation which indicates that large numbers of mutations accumulate over the years such that by the age of 70 years, each cell in the normal colon would have acquired between 0.65 and 2.6 mutations per 100,000 bases (51). At the time that this simple calculation was published actual measurements of the prevalence of mutations in colorectal tumors had reported rates of < 1 mutation per 100,000 bases (52, 53). In the subsequent few years advances in technology have enabled an even more extensive analysis of the actual mutation prevalence in tumors according to the age of the patient. Whole exome or whole genome sequencing data analyzed in tumors from 6,969 patients with 34 different cancers revealed an increase in mutations with the age of the patient (54) and with a prevalence that was within the same range as that calculated for intrinsic mutations in normal tissues (51). Indeed a few studies have now compared mutations within tumors and matched non-malignant tissue from the same individuals and have found the prevalence to be essentially the same (42–44). It therefore now seems likely that most of the common epithelial cancers could well be initiated due to this natural intrinsic mutation rate. Updated modeling of how epithelial tumors arise suggest that more than half of the mutations present originated prior to cancer initiation from the intrinsic natural mutations (55).

In addition to measurements of mutation rates within tumors such measurements have also been made in normal tissues from individuals without cancer. A study of mutations in the oncogene p53 in normal cancer-free adults revealed that cells with mutated p53 comprised up to 4% of the entire epidermis (56). A more recent study of the prevalence of mutations in a range of 74 known oncogenic driver genes in normal skin found mutations in 18–32% of normal skin cells with around 140 driver mutations per square centimeter and many clones of cells with multiple oncogenic mutations (57). A similar study of somatic mutations in normal esophagus from organ donors revealed similar high prevalence and also showed the age dependent accumulation of such normal mutations (58). A study of the rate of somatic mutations at a single gene locus in kidney epithelial cells (again obtained from organ donors) found a rate of 0.2 mutations per 100,000 bases in donors from the first decade of life and this increased with age such that for donors in their eighth and later decades the rate was 4 mutations per 100,000 bases (59). Epigenetic and other genetic defects also accumulate with age (60).

The straightforward mathematics indicates that with around 10^13^ cells in the body over a lifespan many millions of these cells will normally acquire sufficient mutations to generate a malignant genotype. An obvious consequence is then that all individuals who reach an advanced age will harbor large numbers of cells with the capacity to initiate a cancer. Thus, although gene mutations clearly initiate cancers it seems clear from the recent findings that the cancer causing mutations are not rate-limiting: in aging individuals there are thousands or millions of cells with cancer causing mutations and it is the context that determines whether these cells progress to initiate clinical cancers. The environment in the tissues within which the cells reside primarily determines this context.

**Figure 2:**
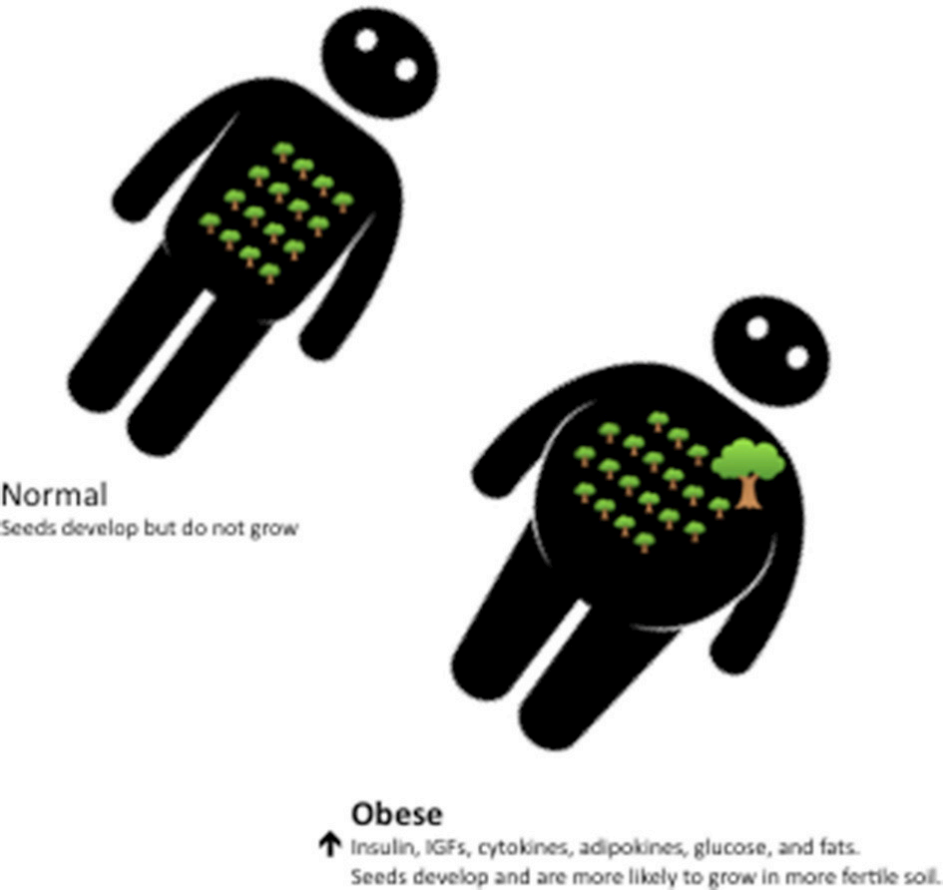
With aging the epithelial cells that have continuously divided throughout life naturally accummulate large numbers of mutations and some of these result in small occult neoplastic lesions, the “seeds.” In normal individuals such lesions develop slowly and rarely progress to clinical cancer. In obese individuals the internal milieu, or “soil,” is characterized by high levels of glucose, insulin, IGFs, inflammatory cytokines and adipokines and this environment increases the risk that latent neoplastic lesions progress more rapidly and develop into clinical cancers.

Well over a century ago Stephen Paget suggested that what determines where a particular cancer spread within the body was dependent not only on the disseminated tumor cells, the “seed,” but also depended on the properties of the tissue where the secondary metastasis occurs, the “soil” (61). Applying a similar analogy for when mutated epithelial cells actually develop into a clinical cancer it would appear that the seeds are not limited, but are increasingly prevalent with age, and that it is the fertility of “soil” that determines whether these plentiful “seeds” actually develop into clinically relevant tumors. The availability of metabolic fuels and the metabolic signals that regulate this availability are then critically important components of this environment as they determine the fertility of the “soil” (Figure 2). That a malignant genotype alone is insufficient to cause a cancer, but also depends on the context, is supported by many other lines of evidence that we have reviewed previously (60). This includes evidence from experimental models in which the same cells are transplanted into different tissues of an animal but only form tumors in some of these and also evidence from human cancers that are caused by somatic mutations that occur in every cell but which only give rise to cancers in very specific tissues (60).

One other consequence of the high normal accrual of mutations that occurs with every cell division is that as cancers grow and spread they should become more and more heterogeneous. Indeed, by the time that a human tumor is clinically apparent or detectable by imaging techniques it has to be already comprised of millions of cells that would have grown over many years or decades since initiation and in this time, it would have accumulated a large number of additional mutations. The mutational evolution of cancers has been predicted for many years but with recent advances in sequencing technologies the actual scale of the heterogeneity has become clear. An early study with extensive sequencing of multiple biopsies obtained from four patients with renal carcinomas enabled a phylogenetic reconstruction of the evolution of these tumors (62). This study found many mutations, some of which were shared across all regions of the primary tumor and metastatic lesions and obviously occurred before the spread of the cancer to the other sites, others were shared across the different regions within the primary but were not present in the metastatic lesions while others were shared within metastatic lesions but were not present in the primary and these had presumably occurred after the spread of metastasis; but there were also many “private” mutations that were only found at one site either within the primary or metastatic lesions and had occurred most recently (62). A phylogenetic reconstruction following the examination of 21 primary breast tumors concluded that a common ancestor clone appeared surprisingly early with considerable subsequent sub-clonal diversification and evolution and that more than 50% of the cancer cells were from a dominant sub-clone that separated from the most recent common ancestor by many hundreds or thousands of point mutations (63). The appearance of this dominant sub-clone appeared to enable growth to the size that became clinically detectable. Metastatic spread, rather than the primary tumor, is the cause of more than 90% of cancer deaths. A recent study examined the phylogenetic evolution of multiple metastases in 10 men with prostate cancer and found that in addition to dissemination of cells from the primary tumor, metastasis to metastasis spread was common and in half of the cases there was transfer of multiple clones between metastatic sites (64).

In addition to the genetic heterogeneity there are several other sources conferring further heterogeneity that can occur even within genetically identical cells. There has been much interest in a sub-population of cancer stem cells, tumorigenic cells that can self-renew; however, there is now considerable evidence that these stem-like cells form part of a broader phenotypic plasticity within populations of epithelial cells. Several reports indicate that not only can stem cells divide asymmetrically to generate differentiated cells but that differentiated cells can also de-differentiate to establish new stem cells (65, 66). Indeed there is evidence that such dynamic interconversions have occurred within actual clinical tumors (67). The factors governing this inter-conversion are starting to be elucidated. An examination of such switching in human melanoma cells implicated the Wnt and PI3K pathways (68). The stem-like cells are not a distinct cell type but just one of a number of different stable differentiation states that cells can switch between. Another important aspect of this phenotypic plasticity is the inter-conversion termed EMT and the reverse mesenchymal to epithelial transition (MET). The most common human cancers are those of epithelial cells that form the barriers within the body and in which cell divisions continue throughout life in order to maintain these barriers but as a consequence also result in an accumulation of genetic alterations due to the cumulative cell divisions. In order to function as a barrier the epithelial cells tightly adhere to each other via adhesion complexes within which E-cadherin plays a central role. E-cadherin not only serves as an important adhesion factor but via its interaction with β-catenin acts as a gatekeeper maintaining the cell differentiation status. Signals such as those from growth factors, that are often increased in tumors, result in modifications to the E-cadherin/β-catenin complex destabilizing adherens junctions and leading to β-catenin translocation to the nucleus where, with the Wnt pathway, it participates in activation of transcription factors that promote EMT (69, 70). This phenotypic switch results in loss of the tight cell-cell adhesion characteristic of epithelial cells and gain of a mesenchymal phenotype characterized by increased motility and invasiveness; facilitating the progression to metastatic disease. Accumulating evidence indicates a role for the insulin/IGF-axis in regulating EMT (71, 72). In relation to this epithelial plasticity two recent studies have also both reported that activation of PI3K can result in de-differentiation of lineage restricted mouse mammary epithelial cells into multipotent stem-like cells that can then differentiate into mixed lineage cells resulting in heterogeneity in subsequent tumors (73, 74).

A further layer of complexity may arise from interactions between tumor cells and host cells. Cancer cells interact within tumors with stromal cells, such as fibroblasts and adipocytes, endothelial cells, and immune cells. In addition to the recent evidence indicating the normal age-related accumulation of mutations within rapidly turning-over epithelial cells it has also become apparent that a normal age-related accumulation of somatic mutations in hematopoietic stem cells results in prelavent clonal hematopoiesis in elderly individuals with mutant clones comprising a large proportion of peripheral blood cells (75). In addition to potentially being precursors for hematopoietic cancers; clonal hematopoiesis has been shown to be a risk factor for age-related atherosclerosis and cardiovascular disease (76). As the immune system plays an important role in both the metabolic disturbance associated with increased adiposity and in policing the spread of neoplastic cells; such clonal hematopoiesis could have multiple potential effects on the progression of cancers.

In addition to, or as a consequence of, the genetic and phenotypic heterogeneity that develops within tumors, as described above, it is also apparent that metabolic heterogeneity also develops, even within tumors (77). Glucose is the main metabolic fuel used in human tissues and Warburg described the switch in metabolism of glucose to meet the energy requirements of growing tumors. However, tissues have evolved a number of different pathways to ensure that essential energy requirements can be met in a variety of metabolic conditions and, unsurprisingly, as tumors evolve they also acquire a variety of mechanisms to maintain their increased energy requirements. In addition to increased glycolysis they can also utilize oxidative phosphorylation and use alternative fuels in addition to glucose, such as glutamine and fatty acids and complex metabolic fluxes can be established within tumor microenvironments between the neoplastic cells and stromal cells such as fibroblasts and immune cells (77). This can greatly complicate the heterogeneity within tumors and strategies to treat them.

The considerable heterogeneity within tumors and metastatic lesions has several important consequences. This heterogeneity provides an explanation for the failure of targeted drugs that generally have shown great between-patient variability in therapeutic responses and only short-term survival benefits. It is unlikely that the same pathways will drive all of the heterogeneous clones within a tumor and therefore even if the dominant clone is successfully targeted, then sub-clones that are driven by alternate pathways would be relieved of the competition from the dominant clone and could then soon regenerate the tumor. This provides an explanation for why most targeted therapies only work for a limited time, until other clones enable the tumor to re-grow. In addition, this heterogeneity will present a considerable challenge to personalized therapy that is based on the genotype/phenotype analysis of a single surgical/biopsy sample. The therapeutic challenge presented by tumor heterogeneity has other implications. In addition to negative interactions between sub-clones, such as competition for resources that may lead to a dominant clone; it has also become increasingly clear that there may be considerable positive interactions resulting from cooperation between multiple clones, such as one clone secreting an angiogenic factor that benefits all clones (78). Interestingly an example of heterogeneous clones sharing “public goods” has been reported with the expression of IGF-II from one clone supporting clonal heterogeneity (41). In addition, the acquisition of drug-resistance associated with tumor heterogeneity has been reported to involve dynamic phenotypic switching with cells transiently acquiring drug-resistance via chromatin-modifications leading to activation of IGF-I receptor (IGF-IR) signaling (79). Such heterogeneity and cooperation may not only favor progression of the primary tumor and drug resistance but may also facilitate metastasis: a sub-clone may have the characteristics that confer an advantage in a secondary site where the ecology may be very different from that in the primary site.

## Cancer Cell Metabolism Rediscovered

In the early twentieth century the advent of modern medicine was based on scientific interrogation; during this time observations of altered cell metabolism in tumors led to this becoming a central focus of cancer research. In the 1920's Otto Warburg put forward a hypothesis to explain the etiology of cancer based on a switch in cell metabolism that he proposed could be the primary defect; an effect that has become known as the Warburg effect (80). Even before this, it was reported that calorie restriction could inhibit the growth of transplanted tumors in mice (81) and in 1926 it was observed that excision of spontaneous tumors in fully fed mice did not prevent the recurrence of cancer in 82% of the animals but that calorie restriction could reduce the recurrences down to 27% (82). Observations on the benefits of calorie restriction subsequently became the most robust intervention for reducing the growth and progression of a large range of cancers in all model systems. These seminal observations placed nutrition and metabolism at the center of cancer research for the first half of the twentieth century. The explosion in molecular biology in the latter half of the twentieth century however relegated altered cell metabolism in tumors to a “Cinderella” status when center-stage became dominated by the focus on genes. The promise of genetic discoveries convinced most investigators that studying the defects in oncogenes and tumor suppressor genes would yield new therapeutic targets that would revolutionize cancer treatment. Despite huge advances in understanding, this paradigm has however largely failed to live up to its early promise. The lack of impact of the prevailing paradigm on clinical disease has led to many questioning the need for a new approach (83, 84). At the same time it has become clear that the global epidemic in obesity and the associated metabolic syndrome and type 2 diabetes are having a big clinical impact on many of the common cancers. This has all contributed to a “re-discovery” of the important role that metabolism plays in cancer. The discovery that a number of oncogenes and tumor-suppressor genes have direct effects on key metabolic pathways helped propel altered metabolism back into the mainstream and was then belatedly added to the “hallmarks” of cancer (85, 86).

Alongside the growing evidence that the obesity epidemic and associated metabolic disturbances may have a big impact on the prognosis of many cancers there has also been more awareness that the widening adoption of a “Western lifestyle” in Asian and African countries has been accompanied by an increasing incidence of some of the most common epithelial cancers. An obvious extrapolation of these observations is that many of these cancers should be preventable. Many studies have observed that migrants, from regions of the world where these cancers are not common, who migrate to Western countries then rapidly acquire the same high incidence as the indigenous population (87, 88). In addition, within Eastern countries that have seen increasing Westernization of diets and lifestyles there has been a subsequent parallel increase in the incidence of these cancers (89). This clearly implies that a reversion of these lifestyle/nutritional effects should provide a means for reducing the incidence of these cancers. The perception that cancer was initiated by unavoidable stochastic genetic defects led many to believe that prevention would be difficult and therefore the only course would be to treat the clinical disease when it is detected. However, in most cases the cancer is well advanced by the stage that it presents clinically. It has been noted that hoping to cure cancer at an advanced stage is as untenable as curing advanced polio or curing a person following a severe stroke (1).

For other widespread chronic diseases, it has long been widely accepted that prevention would have the greatest impact at reducing disease burden at the population level and this has proven the case for cardiovascular and infectious diseases, but thus far not for cancer. This had much to do with how the research agenda had evolved differently in the cancer field, but many have now started to believe that a similar approach could also be successful for cancer. As the prevailing paradigm of multi-hit genetic defects offered little prospect for prevention, this has increased the impetus to explore alternative concepts such as metabolic perturbations. The growing evidence, implying an important role for metabolism, and suggesting that lifestyle alterations may provide a means for prevention has re-invigorated the interest of public health scientists and epidemiologists.

There have been many genetic epidemiology studies of the common cancers; especially Genome-Wide Association Studies (GWAS) that have generally failed to live up to early expectations as there appear to be few common alleles with high penetrance that contribute to these cancers. This has led to studies of larger and larger populations to ensure finding many genes that each had small associations with the cancer (90, 91). At the same time there have been more traditional epidemiology studies examining associations with conventional biomarkers; the most convincing being prospective studies in which biomarkers are measured in blood samples taken from individuals in large healthy populations and these measurements then related to the risk of subsequently developing cancer many years later, thus limiting the potential for reverse causality. The most robust observations made across all of the most common epithelial cancers (breast, prostate, and colorectal) have been associations with insulin-like growth factors (IGF-I and IGF-II) and insulin (4, 92–94). These are markers of metabolic status, that depend on nutrition and lifestyle, implying that they could potentially serve as intermediate biomarkers to monitor the effectiveness of nutrition and lifestyle interventions to reduce cancer. As mediators of interventions that improve metabolic status they could also help to explain how such interventions may affect the course of tumorigenesis.

A cancer develops when cells acquire a growth advantage and lose the normal constraints on growth: cell growth requires substrates for biosynthesis and an increase use of energy to build new cells. In normal quiescent cells, that are not proliferating, metabolism is optimized to extract maximum energy from metabolic fuels. The most available metabolic substrates in the body are glucose and glutamine. The maximum generation of energy from glucose occurs by glycolysis, producing pyruvate which is then fed into the tricarboxylic acid (TCA) cycle in the mitochondria where the carbon is eventually burnt down to carbon dioxide through oxidative phosphorylation. That this may be different in cancer cells was described early on by Otto Warburg who observed that this did not happen in cancer cells but glucose was instead fermented to lactate (80); a process originally thought to only occur in the absence of oxygen restricting oxidative phosphorylation. In cancer cells this however occurs even when adequate oxygen is available and is referred to as aerobic glycolysis. Although this is very inefficient, as it yields a fraction of the ATP per molecule of glucose that could be obtained by mitochondrial oxidation, it was however realized that in proliferating cells rather than burning all the carbon down to CO_2_, the carbon from the glucose was required for the TCA cycle to provide substrates, such as acetyl-CoA, to build all of the macromolecules that were needed for cell duplication. The observed increase in flux through glycolysis is then required to meet the additional energy requirements. Following the observation that this occurs in cancers it was gradually realized that this metabolic switch was a general requirement for most proliferating cells. The same switch to aerobic glycolysis occurs during embryonic development and in cells in normal tissues that continue to proliferate throughout adult life. The switch to aerobic glycolysis that accompanies cell proliferation has been observed in lymphocytes during infection (95, 96) and in epithelia during wound healing (97). It was subsequently recognized to be a requirement for enterocytes, lymphocytes, thymocytes, tumor cells, and indeed all rapidly dividing cells (98).

The insulin/IGF axis evolved to synchronize growth with nutrient availability and unsurprisingly activation of the pathway promotes the metabolic switch required in proliferating cells. The insulin/IGF pathway promotes the Warburg effect by enhancing glucose uptake and via a variety of additional mechanisms. These include stimulating aerobic glycolysis via activation of Akt (99), a switch in the alternative splicing of pyruvate kinase to the less active isoform PKM2 and promoting nuclear translocation of PKM2 via activation of the MAP kinase pathway (100). Promoting the less active PKM2 isoform results in a redirection of glucose metabolic flux from pure energy generation to more anabolic processes (101).

## Selection for Autonomy

The insulin/IGF/PI3K pathway conveys social signals that ensure that cell activities are coordinated according to nutrient availability; if any cells were overactive they may consume inappropriate amounts of nutrients and compromise the viability of other cells. In cancers this social “conscience” is lost and the disease progresses because the tumor develops at the cost of the organism. It is now clear that most tumors are comprised of heterogeneous clones of cancer cells and the most successful clones are those that acquire mutations that convey a selective advantage. As the signal to go ahead and progress according to nutrient availability is a limitation for cells that no longer have concerns for the common good; then acquiring mutations that lead to intrinsic activation of this pathway, and hence become independent of the social constraints, would be selected for as they would confer an advantage. It is now clear that this indeed occurs and mutations in the gene for the p110 catalytic subunit of PI3K, that free it from the restraints of p85, resulting in constitutive activation, are extremely common in a range of human cancers (102, 103). In addition to this, loss of the counteracting phosphatase PTEN, that normally acts as a brake on the pathway are even more prevalent in many common human cancers. Loss of PTEN occurs not just via inactivating mutations but can also be acquired via a number of mechanisms including deletions, transcriptional silencing and protein instability (104). Indeed, loss of PTEN is one of the most common alterations found in human cancers, second only to alterations in p53 (105, 106). This not only indicates the selection advantage, and hence importance, of this pathway to many common human cancers but it also greatly confounds the interpretation of studies of the social regulation of this pathway by insulin/IGFs. Since tumors that have acquired independence of the external cues via these mechanisms will confound studies of associations between the levels and activity of insulin/IGFs and their receptors and they may also confer resistance to therapeutic strategies to block either the ligands or their receptors. Over recent years the true extent of intratumoral heterogeneity has become apparent which further complicates these issues. If just a minor clone within a tumor has acquired independence of these external controls; then even if blockade of insulin/IGFs were effective for the major clones and initially shrank the tumor, it could also add a selective advantage to these minor clones which could then regenerate the tumor. Even an apparent initial response could then lead to resistance to therapy. The effectiveness of blockade of the external insulin/IGF signal would depend on whether acquired constitutive activation of the internal signaling pathway had occurred. If constitutive activation of PIK3CA or loss of PTEN occurred early and a major clone had acquired autonomy, then blocking the external signal may have little effect; if such an event occurred late and only minor clones had acquired autonomy then an initial response may be obtained until these clones repopulate the tumor and then resistance would develop. As most clinical trials are initiated in patients with advanced disease they will have acquired considerable heterogeneity and a range of such responses will occur within different clones.

## Obesity, Diabetes, Insulin/IGFs, and Cancer

Obesity and type II diabetes are the consequence of metabolic imbalances and lead to perturbed levels of metabolic regulators, their mediators, and metabolites. These conditions can impact on the progression of cancers by resetting the context in which the cancer cells are developing both by altering the availability of fuels and nutrients and by altered cues and signals: at least partly mediated by the metabolic regulators. Obesity and perturbed metabolism are associated with altered levels of insulin/IGFs, inflammatory cytokines, and important metabolic fuels. The internal milieu could therefore become a much more fertile “soil” in which malignant “seeds” could develop. There are also complex interactions between these effects: for example, altered availability of lipids and cholesterol are not only potential fuels for the cancer cells but they are also substrates for the local production of steroids that may be important for driving some cancers. In addition, these lipids are also the major substrates and constituents of membrane lipid rafts; domains within the cell membrane that contain the receptors for may signals, including the IR and the IGF-IR, and changes in the composition of these domains can alter these essential cell regulators. Insulin resistance results in increased insulin secretion from the pancreas to try to compensate and maintain glycemic control. The IGF-system is also reset but in a more complicated manner due to the differences in its basic physiology. In terms of circulating levels of IGF-I it is now clear from large population studies that there is a non-linear relationship with low IGF-I levels at both low and high BMI (107–110). Circulating levels of IGF-I increase as BMI increases from low BMI across the normal range into the over-weight range, as expected from the nutritional regulation of IGF-I. With obesity, at higher BMI, however circulating IGF-I levels decrease as hepatocyte function (the main source of circulating IGF-I) is compromised. Consistent with this, weight loss in obese subjects has been reported to result in an increase in circulating IGF-I levels (111), presumably reflecting an improvement in hepatocyte function. Some commentators have concluded that the fall in circulating IGF-I with obesity discounts IGF-I as a major player in mediating the effects of obesity on cancers (112). This however assumes that lower circulating levels equates to lower IGF-activity in the tissues; an assumption that is not sound given our understanding of IGF-physiology. Most of the circulating IGF-I is in the ternary complex with IGFBP-3 and the ALS that is too large to cross capillary barriers. The availability of this to the tissues then involves complex interactions between IGFBPs, proteases and the capillary barrier (10). Genetic manipulations of IGF-I and IGFBP-3 have demonstrated that the biological activity of IGF-I in the tissues is not directly related to the circulating concentration in any simple manner (113). Obesity is also associated with a decrease in pituitary GH secretion but an increase in the production of IGF-I in response to GH probably due to the insulin-induced increase in GH-receptors on hepatocytes (10). With obesity and raised insulin levels there is also a decrease in IGFBP-1 and IGFBP-2 levels and these reductions could potentially result in an increase IGF-availability in tissues (110).

The very indirect relationship between what IGF is active in a relevant tissue and what can be measured in the circulation makes it remarkable that associations between circulating IGFs and cancer have been so consistently observed. It has been commented that the interpretation of such associations between IGFs and cancers in epidemiological studies is greater if the investigators have expertise in the IGF-system than if the investigators are epidemiologists with no prior expertise in IGF-physiology (114). This has been suggested to represent bias; but could just be that those who are knowledgeable of the complexities of the physiology are more aware of the limitations of the circulating measurements and may interpret that an association with such a crude measure of a complex biological system will probably grossly underestimate its real importance.

Obesity and diabetes are increasing in prevalence at an alarming rate and it is now clear that they may have a large impact both on the risk of incidence of a number of cancers and also in promoting the progression of even more cancers (115). The evidence that obesity and diabetes are linked to cancer and the potential causes behind these links has been reviewed extensively over the last few years (116–118). For some cancers there are obvious direct links: obesity clearly involves perturbations in the gastrointestinal system and the main metabolic tissues and glands and these disturbances may directly affect the development of cancers in these tissues. Chronic acid reflux causes damage to the esophagus; gallstones develop in the gallbladder; the liver is damaged by fatty infiltration; pancreatic beta-cell failure and fatty infiltration damage the pancreas. All of these disturbances lead to chronic local inflammation and increase the risk of cancers in these tissues. Sex steroid production is altered via many mechanisms; their availability is increased due to insulin suppression of sex hormone binding globulin (SHBG) and their metabolism is altered, especially due to the high aromatase activity in adipose tissue. All of these contribute to an increase in the risk of cancers that are affected by sex hormone levels; particularly cancers of the breast and endometrium and potentially ovary. In addition to these specific local factors there are clearly systemic factors related to perturbed metabolism that alter the environment in which a tumor may develop. In obesity and diabetes, the availability of metabolites, especially carbohydrates, fatty acids and lipids, are altered; the activity of many metabolic regulators is also perturbed, including insulin and IGFs, and obesity is associated with a chronic low inflammatory state and the levels of many inflammatory cytokines are also affected.

Conventional wisdom suggests that obesity and the development of type 2 diabetes are associated with insulin-resistance and a compensatory increase in insulin levels could then stimulate the growth and metabolism of neoplastic cells via insulin receptors, IR-A or hybrid IGF-I/IR receptors (4, 119). There have however been many inconsistencies with the popular concept that dietary calories, and particularly fat intake, are responsible for obesity; the increasing epidemic has occurred even in populations were total calories or fat intake has not increased and attempts to combat the obesity epidemic with reductions in fat consumption or calorie restriction have failed to make significant inroads. Reduced calorie intake increases hunger and reduces energy expenditure which together lead to weight gain that soon negates any weight loss. An alternative view that has gained traction is that obesity is driven by the increasing abundance of foods with high glycaemic load. Foods such as simple carbohydrates cause rapid, marked increases in insulin secretion resulting in swift partitioning of nutrients into adipocytes with a consequent fall in circulating nutrients which then results in hunger leading to over-eating (120). This has recently been supported by a large study using Mendelian randomization in which associations between natural genetic variants associated with a phenotype and a given outcome are examined. As genetic variants are allotted at conception according to Mendel's second law any association with an outcome is much less prone to confounding or reverse-causality; problems that often limit the interpretation of classical observational association studies. A large population study found that genetic instruments for insulin secretion in response to an oral glucose load were strongly associated with variations in BMI whereas genetic instruments for BMI were not associated with measured insulin response (121). This suggests that variations in carbohydrate-induced insulin secretion drive changes in BMI rather than variations in BMI driving changes in insulin via insulin-resistance. Although normal variations in insulin-response appear to drive normal variations in adiposity: this does not negate the considerable evidence that clearly shows that increases in adiposity cause insulin resistance. Obesity may then result from a vicious cycle in which carbohydrate intake provokes insulin-induced fuel deposition in adipocytes with the increased adiposity then leading to insulin resistance and a further compensatory rise in insulin. The implications of this carbohydrate/insulin model for obesity on the development of cancers is yet to be fully elucidated.

Obesity is accompanied by a large number of systemic changes, in addition to changes in insulin, with increases in IGF-activity, sex steroids, inflammatory cytokines, adipokines, angiogenic factors and metabolic fuels, including glucose, fats and cholesterol. It is clear that all of these factors can affect the behavior of cancer cells and in reality they probably all contribute to altering the internal milieu; changing the internal environment that then affects both the development of the cancer and its response to therapeutic interventions. In tumors with considerable heterogeneity these changes in the internal environment will alter the clonal selection pressures and clones that can take advantage of the increased supply of energy, nutrients and stimulants will gain an advantage.

## The New Genetic Landscape and the Rationale for a Move to Prevention

As described above the last decades have witnessed rapid advances in technology that have enabled the mapping of the genetic landscape of cancers in increasingly fine detail. This has resulted in a growing awareness that the occurrence of large numbers of mutations and the development of occult neoplastic lesions are part of the normal aging process in epithelial tissues. The accumulation of mutations then also continues after initiation of carcinogenesis and can generate considerable intra-tumoral heterogeneity. The new awareness of the etiology of cancers and the problems of targeting therapy has reinvigorated interest in viewing both the “soil” and the “seed.” Observations that alterations in the soil affect the process of carcinogenesis support the proposition that the occurrence of “seeds” is not rate-limiting. Loss of imprinting of IGF-II, in which both paternal and maternal alleles are expressed, increases the risk of developing colorectal cancers (122) and prostate cancers (123) presumably by setting a more growth-promoting and hence more fertile “soil.” Indeed, in a mouse model experimentally induced loss of imprinting of IGF-II in the prostate promoted widespread neoplasic growths (124). Similarly, somatic mutations in immune-related genes can predispose to multiple synchronous genetically distinct colorectal tumors; presumably due to setting an inflammatory microenvironment, or “soil,” that favors tumorigenesis and indicating that the potential “seeds” are naturally present and abundant (125). Obesity is characterized by a chronic low-inflammatory state and could also provide such a fertile soil for tumorigenesis.

The metabolic milieu is one of the most important components of the soil: setting how fertile the soil is for the seeds to grow. The metabolic environment of the tumor is determined by the supply of energy and nutrients and by many critical metabolic regulators: of which insulin and the IGFs play a central role. There has been considerable recent progress in our understanding of how oncogenes, tumor suppressor genes and malignant transformation results in resetting of tumor cell metabolism but there is still much to learn regarding how systemic and local metabolic factors impact on the processes of tumor progression and metastasis. Population epidemiology strongly links the clinical prevalence of the common epithelial cancers to a Western lifestyle. The generally high prevalence of occult cancers in populations however appears to be similar in all parts of the world (60); implying that the impact of a Western lifestyle is not on cancer initiation but affects the progression from latent, occult lesions to clinical disease. There are limitations in interpreting autopsy studies conducted in very different settings due to large variations in methodology but a comparison of occult prostate cancers in black American men with those of white American men by the same pathologists revealed an identical prevalence (126). In both groups the prevalence increased with age, exactly in parallel with the increase in genetic mutations with age (51, 54). The prevalence was the same in black and white men despite the former having a 60% higher incidence of clinical prostate cancer and a 2 to 3-fold higher mortality rate (126). This is consistent with the initiation of tumors being the same in black and white men but more of these progressing to clinical disease in the black men. Similarly, a direct comparison between Caucasian and Asian men revealed a very similar prevalence of occult prostate cancer despite large disparities in clinical disease (127). The same conclusion is implied from a meta-analysis of population studies which indicated that circulating measures of IGF-I were related to the risk of prostate cancer in studies where the cancers had been detected as clinically presenting disease but not in studies where the cancers have been detected by prostate-specific antigen (PSA) screening (which are mainly low-risk cancers that never progress to clinical disease) (128). An even stronger association was observed between IGF-I and mortality from prostate cancer (129). Together these studies indicate that metabolic status, as reflected by IGF-I measures, have little effect on the occurrence of latent, occult lesions, but promote the progression to clinical cancers. More epidemiology is required with more detailed phenotypic data in order to better characterize how exposures affect the different stages of progression rather than just analyzing incidence.

The accumulating evidence implies that metabolic derangements associated with a Western lifestyle, and therefore presumably the metabolic regulators that mediate these effects, act primarily on progression to clinical disease rather than on initiation. Metabolic regulators such as insulin and the IGFs promote the required resetting of cancer cell metabolism and the initial clonal expansion; but then also help sustain the de-differentiation and acquisition of clonal heterogeneity that then favors progression and metastasis. That these fundamental controls are important for cancer progression is supported by the prevalence of genetic alterations to the pivotal PI3K/PTEN signaling pathway that have been observed in tumors across populations. With the considerable heterogeneity that is now known to exist within tumors it is highly likely that similar selection pressure would result in at least some of the many clones also acquiring such alterations. It should then not be too surprising that targeting upstream activators of this pathway showed limited effectiveness when tested in trials in patients with advanced cancers (5,7) since even if the major clone initially responds, the presence of minor clones with autonomous activation of the PI3K pathway could result in ultimate treatment failure. Following the disappointment with this approach there has been considerable interest in targeting the primary metabolic disturbance. There are many effective drugs widely employed to tackle the diabetes epidemic and there are currently a large number of trials examining drugs like metformin against a variety of different cancers (130, 131). If the same development path is followed and these are only tested in clinical trials in patients with advanced disease, who have failed existing treatment options, then it should not be surprising if progress is limited. However, as the evidence suggests that the effect of metabolic milieu is post-initiation, at some stage in the progression to clinical disease, then this is where these interventions may provide the most benefit. Clinical trials in cancer patients at earlier times in the disease pathway are notoriously difficult for many reasons. Identifying meaningful clinical endpoints is difficult and there has been considerable reluctance in trialing potentially toxic new agents when other options may still be available. The increasing emphasis on prevention rather that cure however may provide opportunities for new approaches. This is assuming that it is accepted that prevention means preventing clinical disease and not preventing initiation: as it is evident that for many epithelial cancers initiation may occur as a consequence of normal aging. Drugs such as metformin have an advantage in such a setting as they have a long track record and a well-documented safety profile with limited adverse effects experienced in huge populations treated for metabolic disorders. This is the most promising setting for examining insulin/IGFs; as their robust associations with cancer outcomes indicate that they may be useful intermediate biomarkers. The increased detection of early cancers in population screening programs may also generate suitable target groups for such trials. This is already the case for prostate cancer where increased use of PSA testing has resulted in the detection of large numbers of early cancers. Many studies have indicated that the majority of these are indolent cancers that will not impact on the lifespan of the men (132, 133). Lifestyle changes or interventions such as metformin, that are known to do no harm, would be attractive approaches to examine if they reduce the risk of these indolent cancers progressing to life-threatening disease. However, very few of these cancers progress within a reasonable timeframe (134), making trials with clinical endpoints difficult. Metabolic markers, such as insulin and IGFs, that have established links with clinical cancers are currently being employed as intermediate endpoints. This is supported by the observation that circulating IGF-I levels were associated with evidence of biochemical progression of prostate cancer in a large cohort of men followed for 8 years in an active surveillance program (134). However, eventually hard clinical outcomes will probably be required in such studies. In the future similar studies could also be envisaged in colorectal cancer and breast cancer however these will depend on the establishment of reliable markers of low-risk disease and markers of disease progression. Of particular note, the strategy of calorie restriction that was first observed to restrict the growth of tumors and their recurrence more than a century ago (81, 82) has again become a topic of increasing interest. Several different approaches to calorie restriction are now being investigated at various stages of the cancer pathway and measures of IGFs are the most favored intermediate biomarkers to monitor response (135).

With the realization that by the time that most cancers are clinically evident the acquired heterogeneity and plasticity result in immense challenges for attempts to cure advanced disease, the emphasis for reducing the burden of cancer will increasingly shift to strategies for prevention. Concurrently metabolic imbalance is about to overtake smoking as the leading preventable cause of cancer and this will be the main focus of future studies of prevention. Both lifestyle and pharmaceutical interventions may provide new strategies to slow the progression of most of the common epithelial cancers and this may enable many individuals being prevented from developing lethal disease.

## Author Contributions

All authors listed have made a substantial, direct and intellectual contribution to the work, and approved it for publication.

### Conflict of Interest Statement

The authors declare that the research was conducted in the absence of any commercial or financial relationships that could be construed as a potential conflict of interest.
